# Can Rhesus Monkey Learn Executive Attention?

**DOI:** 10.3390/bs6020011

**Published:** 2016-06-13

**Authors:** Jessica Bramlett-Parker, David A. Washburn

**Affiliations:** Language Research Center, Department of Psychology, Georgia State University, Atlanta, GA 30302-5010, USA; bramlett1@student.gsu.edu

**Keywords:** attention training, rhesus monkeys, executive attention, Stroop task

## Abstract

A growing body of data indicates that, compared to humans, rhesus monkeys perform poorly on tasks that assess executive attention, or voluntary control over selection for processing, particularly under circumstances in which attention is attracted elsewhere by competing stimulus control. In the human-cognition literature, there are hotly active debates about whether various competencies such as executive attention, working memory capacity, and fluid intelligence can be improved through training. In the current study, rhesus monkeys (*Macaca mulatta*) completed an attention-training intervention including several inhibitory-control tasks (a Simon task, numerical Stroop task, global/local interference task, and a continuous performance task) to determine whether generalized improvements would be observed on a version of the Attention Network Test (ANT) of controlled attention, which was administered before and after the training intervention. Although the animals demonstrated inhibition of prepotent responses and improved in executive attention with practice, this improvement did not generalize to the ANT at levels consistently better than were observed for control animals. Although these findings fail to encourage the possibility that species differences in cognitive competencies can be ameliorated through training, they do advance our understanding of the competition between stimulus-control and cognitive-control in performance by nonhuman and human primates.

## 1. Introduction

Executive attention is the active, intentional selection and processing of a stimulus or response. Schneider and Shiffrin [1] summarized the use of this type of controlled processing as serial, flexible, requiring working memory, and relatively unpracticed. This is the opposite of automatic processing, which is highly practiced, rapid, stereotyped or rigid, and which operates without demands on attention, cognitive control, and working memory. Many theorists (e.g., [2,3,4]) have described executive attention as endogenous, or goal driven, requiring the use of top-down processing. These theorists distinguish such control of attention from exogenous, which uses bottom-up processing and is stimulus driven, such as reflexive, elicited, or captured attention. Thus, one can view executive attention as willful, effortful control over selection for processing, particularly under circumstances in which attention is being attracted elsewhere by lower-level mechanisms.

The study of attention has a long history within psychology, as indeed has the notion of controlled or executive attention. William James [5] (p. 404) said that, “everyone knows what attention is”; however, the construct itself has proved more complicated than a simple definition might suggest. This complication led to attention researchers’ and theorists’ frequent appeal to metaphors to facilitate the definition of attention. Attention has been likened to a bottleneck that restricts the processing of multiple, simultaneous stimuli, such that some but not other information becomes selectively processed [6,7]. It has been compared to a filter that blocks some stimuli from being perceived or some responses from being executed [6,8,9]. Attention has been defined as an attenuator or volume-control [10] that predominantly amplifies the most salient stimuli for processing, though other stimuli may be processed at a reduced level as if the volume were lower. Attention has been considered to be a resource [11,12], like a mental energy that can be expended, such that more energy or resources means more attention. For more than a decade, scholars debated the nature and number of these attention resources. Researchers [2,13,14] have also examined attention as if it were a spotlight that shines on interesting stimuli. Other scholars consider attention to be the glue that fuses perceived features to recognizable objects [15]. Neisser [16] compared attention to apple picking, such that attended objects are like apples that are picked from a tree, and unpicked apples are likened to information that is simply not processed. Neisser also wrote that the intentional attention to material, like picking the most desirable apples from a tree, is a skill that can be improved.

At issue is that each of these (and other) metaphors accurately capture some but not all aspects of attention. That is, most of these metaphors do not take into account that there may be different kinds or functions or components of attention. In an effort to make sense of the various models proposed, attention theories have been placed into categories such as early- or late-selection accounts, referring to when stimuli are selected for processing; or cause or effect categories, which are distinguished by whether attention determines or is determined by selection for processing. In light of these theoretical debates and all the research that has been published since James’ [5] famous sentiment, we might conclude that no one *really* understands what attention is and how it works.

To assess the various aspects (or components, or types) of attention, an Attention Network Test (ANT) was developed, and has been successfully used with adults and children (e.g., [17]). Incorporating both a flanker task and a cued reaction-time task, and following the logic of the Stroop task, the ANT showed stable performance within normal adults and produced reliably orthogonal measures of the executive attention, alerting, and attention-orienting [18]. In that study, repeated testing using the ANT reduced participants’ overall reaction times, but had little impact on the derived measures of executive attention (*i.e.*, the difference in response time between incongruous and congruous conditions), making it an excellent measure of attention skills.

Using the ANT as the primary outcome measure, Rueda and colleagues [17] created an attention training intervention using computerized tasks designed for 4- and 6-year-old typically developing children. This intervention was based on tasks developed by Rumbaugh, Washburn and colleagues [19,20] to train nonhuman primates to respond to computer-generated stimuli. Anecdotally, these monkeys seemed to become more attentive in the course of such training, and Rueda and colleagues tested whether similar tasks might systematically affect the attention skills of young children. In 5 sessions, participants completed anticipation, stimulus discrimination, and conflict resolution exercises to correspond with alerting, orienting, and executive control networks respectively. Children were given the ANT and a standard intelligence test before and after testing. Children in the intervention group showed significantly improved executive control scores as well as improved intelligence scores compared to the children who did not receive the intervention. An overall improvement in reaction time was also shown, although significant overall error reduction was absent. Additionally, electrophysiological recordings showed increased maturation in participants following training, suggesting that neurological changes resulted from the training as well.

Research investigating attention training in participants diagnosed with clinical disorders such as Attention Deficit and Hyperactivity Disorder (ADHD) indicates that attention training may reduce ADHD symptoms. Kerns, Eso, and Thompson [21] conducted a study in which 14 ADHD-diagnosed children from the ages of 7 to 11 years were given an intervention called “Pay Attention!” that included materials designed to train attention. Significant improvements were seen on many of the post-test measures, including a version of the Stroop task and an academic efficiency measure. However, significant improvements were also seen in the control group, albeit not to the same extent as the treatment group.

In another study to determine the effects of attention training, Shalev *et al.* [22] tested children diagnosed with ADHD from 6- to 13-years-old using a computerized progressive attention training (CPAT) program. The program provided individualized training specific to each network of attention. A Continuous Performance Task (CPT) was used in an attempt to improve the function of sustained attention, whereas a Conjunctive Search task was utilized to improve selective attention. A combined orienting and flanker task was designed to improve orienting attention, and a Stroop-like task was used to improve executive attention. The intervention was conducted over 8 weeks for 1–2 h each week. Compared to age-matched controls, the experimental group showed significantly reduced inattention, based on behavior scales completed by parents, and significantly improved academic scores.

Klingberg, Forssberg, and Westerburg [23] administered another form of cognitive training that focused primarily on working memory as an outcome. ADHD-diagnosed children between 8- and 14-years of age participated in a computerized, adaptive training intervention. The intervention consisted of working memory span tasks (remembering the position of objects in a 4 × 4 grid) as well as verbal tasks (remembering phonemes, letters, or digits), automatically adjusted for the span of each child. Control participants were given the same tasks but without the adjustment mechanism, so that they stayed at the same low difficulty level throughout. Experimental participants showed significant gains on digit span, visuo-spatial working memory task, and Stroop measures. Restlessness was also monitored, and the intervention group showed a 74% decrease in this measure relative to baseline.

Diamond, Barnett, Tomas and Munro [24] conducted a study to determine the effect of an intervention created to enhance executive function skills in preschool children. Aptly named “Tools of the Mind,” this intervention used several classroom-based activities over the course of a year or more that promoted executive functions. Compared with children in the control group, children who received Tools of the Mind training showed impressive gains in the researcher-defined core areas of executive functioning: inhibitory control, cognitive flexibility, and working memory. Children in the experimental condition that were exposed to Tools of the Mind significantly outperformed control participants on all measures of executive functioning, especially those requiring the kind of inhibitory control that seems important for executive attention.

Evidence shows that attention capacity can be improved across different age ranges and populations. Given that children’s attention and executive functioning skills show improvement with specific training, it seems reasonable to ask whether similar training might improve the executive attention skills of nonhuman primates. Indeed, discovering how to improve executive functioning in nonhuman primates may provide valuable insight regarding the fundamentals of executive control training, which would in turn enhance our knowledge of how to improve these skills in humans. However, this may not be as simple as it sounds due to the fact that nonhuman primates have shown particularly poor executive-attention skills [25]. In fact, some have argued that executive functions (including executive attention and other future-oriented cognitive competencies) are a primary cognitive difference between humans and nonhuman animals (e.g., see discussions in [26,27]).

There is some evidence supporting that nonhuman primates have similar attention mechanisms to humans [28,29,30,31], but rhesus monkeys seem to have less ability than human adults to control attention executively or intentionally. In a study conducted by Washburn [32], reaction time data showed Stroop-like interference in rhesus monkeys comparable to interference experienced by humans. Rhesus monkeys (*Macaca mulatta*) and humans were given a computerized task in which they had to choose the larger of two arrays based on the number of items, not the value of the numbers comprising the array. Because of their learned knowledge of number values, the monkeys had significantly longer reaction times for incongruent trials—trials in which the number of items and the value of the number containing the array differed—than baseline or congruent trials, in which the side with the most was also the side with the largest numeral. The results overall were comparable to the results garnered from human participants in the same study. However, the monkeys were less able to control their attention than humans. Moreover, manipulations of the task that emphasize the need for executive *versus* stimulus control of attention magnify the differences between humans and macaques [33]. For example, reducing the ratio of incongruent-to-congruent trials increases the difficulty of executive attention; under these conditions, the differences between humans and monkeys are even larger with respect to Stroop-like interference. Manipulations of incentive improve performance for both species, but result in significant reductions in interference effects only for humans. This general pattern of species differences in executive-attention performance has been replicated using other tasks (e.g., [30]), each showing that human adults outperform rhesus monkeys under conditions that specifically require attention control in the face of strongly competing stimulus and associative cues.

If the monkeys’ (relatively poor) ability to control attention contributes to their poorer performance on executive attention measures such as Stroop and the executive attention portion of the ANT, and given that children who completed attention training showed the greatest improvement in executive attention more than other attention factors, then monkeys’ attention control ostensibly may be improved by executive attention training. The similarities between human and monkey neural responses to executive-control tasks support the theory that monkeys should perform similarly to humans on an attention-training paradigm. Therefore, if the model of traditional cognitive rehabilitation is used, attention ability should be improved by extensive practice on attention tasks. To be effective, an executive-attention training program or intervention should include inhibitory control tasks. Inhibitory control tasks require an intentional suppression of a strong response tendency. Increasing the ability deliberately to suppress such tendencies should lend itself to increased executive attention ability because inhibitory control is a primary component of executive attention.

To test this hypothesis, a preliminary study was conducted in which task-savvy rhesus monkeys were given 10 computerized blocks of 1000 trials of a spatial incongruity task for executive attention training [34]. Each block of 1000 training trials was followed by 100 trials of a numerical Stroop task to determine whether improvements in attention were specific to the training task or would generalize to other tasks requiring the same cognitive processes. Reaction time on the spatial incongruity task improved across blocks, suggesting improved inhibitory ability; however, generalization to the other attention task, Stroop, was not observed. This indicates that the improvement in the spatial incongruity task came from specific stimulus-response changes rather than from a general improvement in executive functioning or attention-control skills. More specifically, the spatial component of the task became less potent than the learned responses to the stimuli.

Monkeys perform similarly to humans on the Attention Network Test (ANT) as well. In a study conducted by Beran and collaborators [35], children and rhesus monkeys were given a modified version of the ANT. Flankers on either side of the stimulus were either dashes (neutral condition), or the same (congruent) or opposite (incongruent) letter. Both children and monkeys demonstrated significantly faster response times to neutral flankers than to congruent or incongruent flankers. However, children showed significantly faster response times to congruent flankers than incongruent flankers, a result that was paralleled by one of the two monkeys in the study. These results raise more questions about whether or not it is the difference in capacity to attend overall that makes the difference between primates and humans, or the ability to control the attention.

The reason that generalization did not occur in Washburn’s training study [34] is unknown, but it stands to reason that 10,000 trials of one task might lead to associative learning. Regardless of the reason, this improvement demonstrates that monkeys can get better at inhibitory-control tasks, but perhaps some variation in training is needed to reduce the risk of specific stimulus-response conditioning and promote generalized, relational learning. Indeed, using a larger number of tasks that involve conflict between stimulus cues should generalize learning, rather than leading to performance changes that are simply the result of conditioning. Therefore, we expect that when given sufficient training and exposure to multiple inhibitory control tasks, subjects will show significantly greater executive attention as measured by the ANT.

## 2. Method

### 2.1. Participants

The participants were four adult male rhesus monkeys (*Macaca mulatta*) housed at the Language Research Center of Georgia State University. The monkeys were not food or water deprived at any time during the study, or reduced in body weight for purposes of testing. The nonhuman primates (hereafter primates) involved in the study were familiar with joystick manipulation used with computerized testing prior to the present study and have long and well-documented histories of testing in cognitive studies [19,25,36]. Some of the animals had been trained or tested on some of the tasks in this study, whereas other tasks were new for these animals.

### 2.2. Apparatus

Training and testing took place in each monkey’s home enclosure, each of which is attached to a computer-based automated test station (which we have called the “Rumbaughx” [37] to recognize Duane Rumbaugh’s role in the initial idea, and to put the apparatus in historical context with devices like the puzzle box, shuttle box, and Skinner box) that includes a computer, monitor, joystick, pellet dispenser, and external speaker. Stimuli were presented on the monitor, and subjects responded by manipulating the joystick.

### 2.3. Tasks

The computerized tasks used in this study had many common features. Irrespective of task, each trial began with a trial-initiation procedure in which the animal moved a cursor into a circle that appeared in the middle of the screen. Across tasks, correct responses resulted in auditory feedback and automatic delivery of a fruit-flavored chow pellet, whereas incorrect responses resulted in a buzzing noise and a 5-s time-out period.

#### 2.3.1. Generalization Task—Attention Network Test (ANT) 

**Figure 1:**
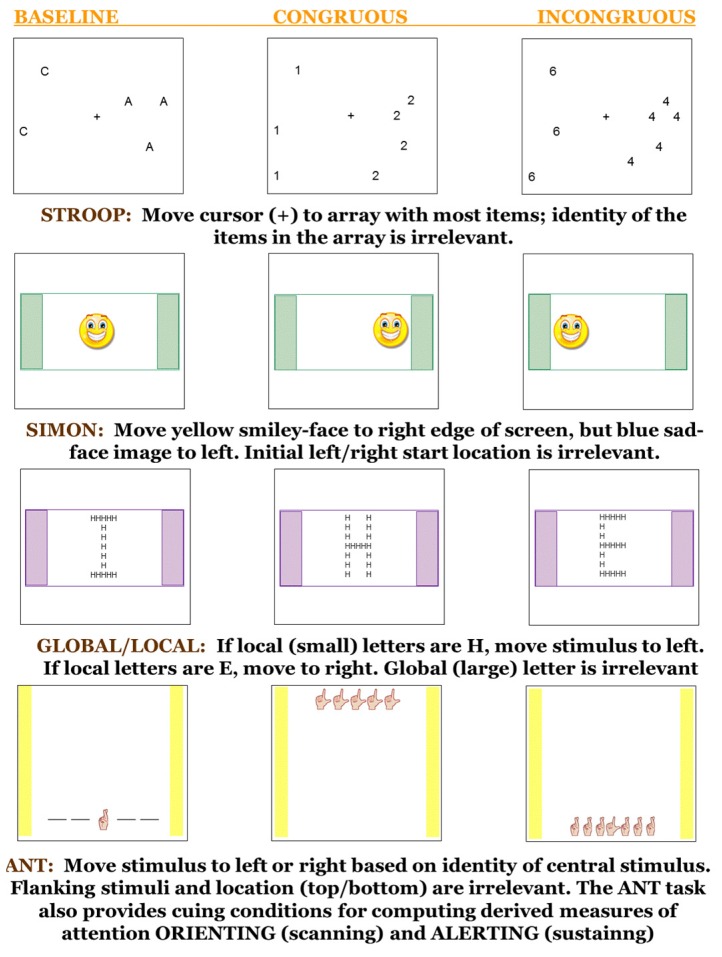
Schematic for the tasks used in this study.

The ANT is designed to provide separate, orthogonal measures of attention orienting, alerting, and executive attention in human adults and children [17,18]. Used widely to determine the efficiency of attention networks, the test requires a participant correctly to identify whether a stimulus requires a left or right response. In the typical version for adult humans, the stimulus is a centrally-located, left- or right-facing arrow. Across trials, the target arrow is flanked on either side either by neutral stimuli (baseline condition) or by arrows that point in the same direction (congruent condition) or the opposite direction (incongruent condition). On some trials, the location of the target + flanker stimuli may be cued so that participants can anticipate when and/or where the arrows will appear. Reaction times and accuracy are recorded and compared across conditions to determine attention efficiency for each of the three attention networks. The test has been modified using different target and flanker stimuli for use with children and monkeys [17,35]. For the present study, we used images of a drawn human hand demonstrating the sign-language letter L or the finger-spelling letter R as the target and flanking stimuli (see Figure 1, bottom panel). These novel stimuli were chosen to lessen potential carryover effects of any prior training, and in recognition of the monkeys’ inability to distinguish left-right mirror-image stimuli [38] like those commonly used in ANT studies with human participants. On cued trials, a large asterisk was presented for 100 ms before vanishing and being immediately followed by the target + flanking stimuli.

The monkeys were trained to deflect the joystick to the left in response to the L image or right in response to the R image. When the monkeys deflected to either side, the center stimulus moved to the corresponding edge of the screen. This emphasized the direction that the stimulus should move as well as demonstrated to the monkeys that the center stimulus is the only meaningful part of the stimulus array. Additionally, correction trials were used during training so that the animal could not progress until the trial was completed correctly. These repeated or correction trials were not included in calculation of performance.

#### 2.3.2. Intervention Task—Modified Simon

**Figure 2:**
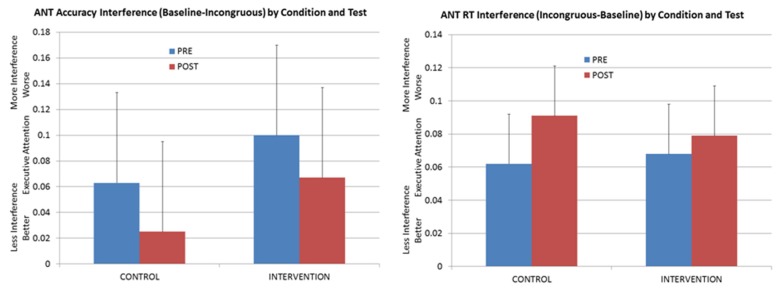
Mean ANT performance (difference between baseline and incongruent conditions) as a function of group (control *versus* experimental or intervention) and test (pre-training, post-training).

The Simon task utilizes the tendency to respond toward the source of the stimulus to measure reaction times to both spatially congruent and incongruent stimuli [39]. In this study, the monkeys were taught to deflect the joystick to the right to move a yellow smiley-face stimulus to the right border of the screen, and to respond left if a blue sad-face appeared as the stimulus (see Figure 2, second panel). Stimuli could initially appear in a neutral position (midscreen) or in a position that was either congruent (e.g., the yellow face on the right side of the screen) or incongruent (e.g., the yellow face on the left side of the screen) with respect to the required response. Note that response time was recorded from stimulus appearance to joystick deflection, so that it was not confounded with initial position or distance to the edge of the screen.

#### 2.3.3. Intervention Task—Continuous Performance Test (CPT)

As its name implies, the Continuous Performance Test (CPT) requires participants continuously to perform a go/no-go discrimination and to remain vigilant as a sequence of stimuli appears on the screen. Although the CPT can be used to study sustained attention (*i.e.*, to examine changes in attentiveness over a long watch period), it was used in the present study primarily because it requires participants to inhibit a prepotent response to a no-go stimulus, where inhibition is made difficult by embedding the no-go image in a series of “go” responses to other stimuli. Our CPT begins with a midscreen cursor and four walls as targets. When a trial was initiated, one randomly selected border of the screen turned blue and the monkey had to deflect the joystick in the corresponding direction (e.g., move the joystick handle down if the bottom wall had turned blue). If this was done correctly, then another randomly determined border would immediately turn blue, and the monkey had to respond accordingly in one of the other three directions. This series of directional responses continued as different borders of the screen illuminated in blue; however, occasionally a border would turn red instead. If the monkey deflected the joystick in the direction of a red wall, the trial immediately ended with a buzz and no reward. Rather, the monkey was required to refrain from deflecting the joystick for 3 s when this no-go stimulus appeared, whereupon the trial ends with a tone and reward delivery. Each CPT problem thus consisted of 5 to 15 responses, and ended either (a) by an incorrect response to a blue stimulus; (b) by a response to a red stimulus; or (c) by 3 s of nonresponse to a red stimulus.

#### 2.3.4. Intervention Task—Numerical Stroop 

The monkeys in the present study have been trained previously to respond to the relative values of Arabic numerals, such that they can choose the larger of two or more numerals so as to receive the corresponding number of pellets [40]. Whether such performance involves knowledge of the values represented by the numerals or simply differential response strengths corresponding to the relative reward magnitude associated with each numeral, this training serves as the foundation for the numerical-Stroop task used here. In the well-known Stroop color-word task [41], participants respond to the color of words and try to ignore, if possible, the incongruent meaning of some of the words. In the numerical version of the Stroop task (see Figure 1, top panel), participants were rewarded for selecting the larger of two arrays based on the number of items, where those items might be letters of the alphabet (e.g., five As *versus* two Cs; neutral or baseline condition) or congruent (e.g., six 4 s *versus* two 1 s) or incongruent numerals (e.g., seven 2 s or five 3 s). Thus, on incongruent trials the animals had to inhibit the response tendency associated with the larger numeral and respond instead to the array-size dimension [32,33].

#### 2.3.5. Intervention Task—Global/Local Interference (GLI) Test

The global/local interference (GLI) task [42] consists of responding to hierarchically-organized images, created by constructing a large figure out of many small ones (see Figure 1, third panel). For example, a letter H made of many small Es was one incongruous GLI stimulus. Upon trial initiation, a large, hierarchically-organized letter was presented in the middle of the screen. For congruent trials, both the large (global) and small (local) letters were identical. Participants will be required to deflect the joystick to the right if the local features were Es, or to the left if the constituent elements were Hs, irrespective of the overall pattern. Thus, the monkeys had to inhibit influence from the global dimension and respond instead to the local features.

#### 2.3.6. Control Test—Horizontal/Vertical Illusion (HVI) Task 

In the Horizontal/Vertical Illusion (HVI) task, the monkeys were presented with a trial initiation screen as outlined previously, followed by a fixation cross for 500 ms, followed by a horizontal and vertical line stimulus (an inverted T). The length of the horizontal line remained constant across trials; but vertical-line length varied across trials. To obtain rewards, the monkey was required to indicate which line was longer, the horizontal or the vertical, by dragging his selection to the corresponding edge of the screen (*i.e*., left if the horizontal line were longer, right if the vertical line were longer). Note that this task is in many ways similar in appearance and response to the intervention tasks, but there was no incongruent condition with strong competition from a to-be-inhibited response cue.

### 2.4. Procedure

As summarized in Table 1, the general procedure for the study was for the monkeys to receive training to asymptote on the ANT task, then to be assigned randomly to either the experimental or control group, and then to complete training and testing on the suite of intervention tasks (experimental group) or control task (control group) before being tested for generalization on a post-intervention ANT administration. One of the control animals was then tested in the experimental condition, and one of the experimental animals then completed the control-task training and testing. Finally, all animals completed the ANT assessment again. The table below illustrates the condition sequence for each of the four monkeys.

In the pre-training assessment, monkeys performed 1000 trials per day on the ANT test, administered along with multiple other tasks available to the animals each day as part of other, unrelated studies. Once an animal reached asymptotic baseline, he was moved to the executive-attention or control training. In the executive-attention condition, the animals were tested on blocks of Simon, Stroop, GLI, and CPT each day (again, along with other tasks from other studies not specifically related to attention). Similarly, the control-group animals received the HVI task along with other computerized tests from other experiments each day. Once the control or experimental tasks were completed the monkeys were again given 1000 trials of the ANT.

## 3. Results

Table 2 shows relevant means for the executive-attention assessments in this study. Across animals and conditions, accuracy was typically better (except in cases of ceiling effects) and responses were typically faster in the baseline than the incongruent conditions, as would be predicted for these and any tasks in which there is strong response competition across the stimulus cues.

Of greater interest for the purposes of this study, however, is whether interference on these incongruent trials was reduced as a function of training and practice. The general answer to this question is “yes” as indicated in Table 3, which summarizes the correlations between trials and interference.

Note that, across animals, difference between baseline- and incongruent-trial got significantly closer on the Stroop and Simon tasks. Note that this reflected improvement in the incongruent-trial performance (*i.e.*, reduction of interference)—an effect that Table 3 shows was significant for Stroop, with positive but nonsignificant correlations (0.16 and 0.37) for Simon and GLI as well—rather than a progressive trend toward poorer performance in the baseline condition. This is important, because it would be unreasonable to expect generalized improvements in executive attention if there were no localized benefits of practice on these attention-demanding tasks. However, the opposite pattern of effects was generally observed on the ANT task. Across days, the incongruent condition tended to be even more deleterious to performance, irrespective of intervention condition.

It is performance on this latter assessment that is most critical for the present study, as it served both as a generalization test and also as a way to tease apart the executive-attention intervention from any simple improvement that occurred (even in control animals) from repeated administration of the ANT. As is evident in Figure 2, no consistent pattern of change was observed in ANT interference. Comparing pre-intervention with post-intervention ANT performance across the two groups (experimental and control) shows that interference declined with training with respect to ANT accuracy, but interference actually increased with respect to the response-time measure of incongruent-trial interference. Further, for the accuracy data, the pattern of improvement that was observed for the animals in the experimental group was not reliably different than the improvement that was achieved by the control group. Thus, this improvement likely reflects the benefits of additional exposure to the ANT task itself rather than to any generalized improvement in executive attention as a result of varied experience on a small suite of inhibition tasks.

**Figure 3:**
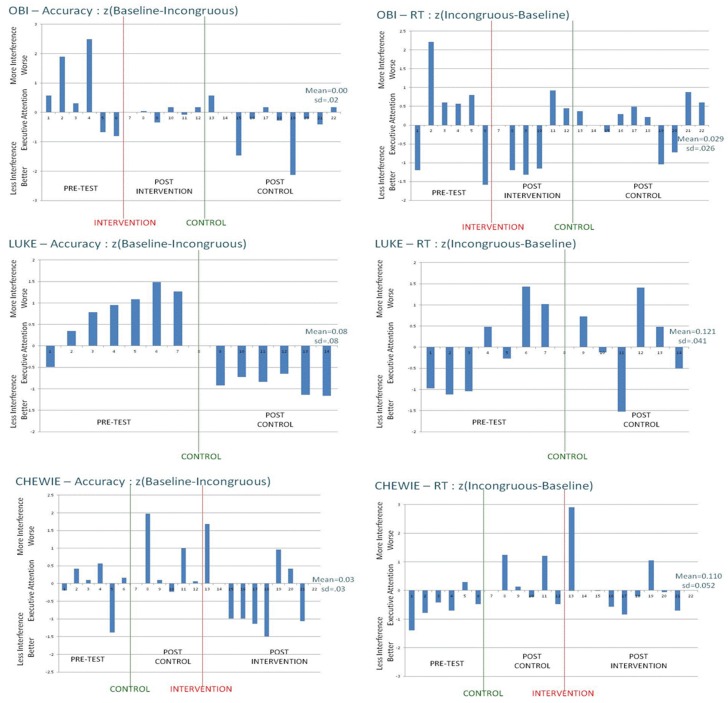

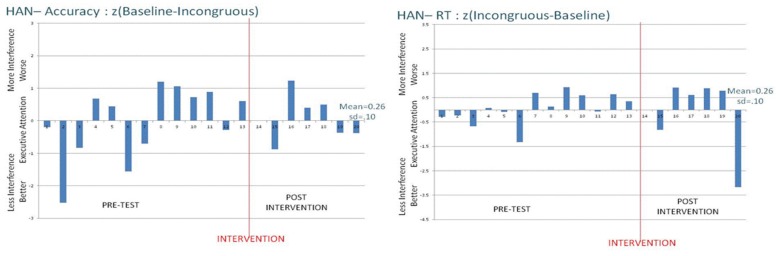
Standardized differences between baseline and incongruous conditions on the ANT task for accuracy (*left* panels) and response-time (RT; *right* panels) measures, by animal (row), test block, and experimental condition.

This conclusion is further supported by the detailed pattern of results across the four individual animals in this study, shown in Figure 3. If training produced generalized benefits for executive attention, then one would expect systematic patterns in the direction and extent of the bars in Figure 3—perhaps like Luke’s change in accuracy measures across days (*i.e.*, large ANT interference effects disappear, suggesting improved executive attention after the intervention). However, Luke was a control-task animal rather than being in the attention-intervention condition. Across animals and measures, Figure 3 shows the great variability in interference effects across days, and the unreliable pattern of change as a function of intervention conditions. Although these monkeys did learn to do the tasks, did generally benefit from training and practice, and did tend to show the expected interference effects that are characteristic of executive-attention tasks, there is nothing in the present results to compel the conclusion that rhesus monkeys can learn to be more executive or cognitive in control of attention.

## 4. Conclusions

There is no question that performance can be improved with training and practice; however, it is less clear whether such training can change cognitive capacity. That is, are there types of training that do more than move an organism toward the most accurate, rapid, efficient level possible for that individual, but that moreover increases the optimal level of performance that is possible for that particular organism? This is the debate that has ranged in the education and intelligence literatures for decades, and that has more recently become a target of considerable attention in the working-memory capacity and cognitive-training domains. For the present purposes, generalized improvements in performance that serve to reduce species differences in cognitive competencies would help to inform both the nature and the causes of those differences. Interventions that prove to be effective in improving controlled-attention skills in nonhuman animals, which are highly susceptible to distraction and stimulus control, would then be good candidates for implementation with human children and adults who struggle with attention-control issues. Thus, there are many reasons to be motivated to find ways of improving the cognitive control of attention in nonhuman animals that have demonstrated deficits, at least compared to human adults, in this area.

Nevertheless, the present findings corroborate our earlier efforts [34] in that training results in localized improvements in task performance but little or no evidence of generalized benefits that would be indicative of underlying changes in cognitive competence. There are several potential reasons that this intervention failed. We had hoped that training monkeys on multiple inhibition tasks would provide diverse practice on executive attention, and thus be more likely to generalize than were our previous efforts with just a single training task. This intuition, which stems from abundant literature showing improved generalized or relational learning with large *versus* small sets of training stimuli, may well be exactly right, but the number of intervention tasks we selected might still be too small to produce generalized effects. This possibility is further encouraged by an examination of our GLI task and data. A software error resulted in the animals being rewarded for discrimination of the local features (global irrelevant, as described above); however, rhesus monkeys, unlike human adults, have a local bias and thus would have found it more challenging to respond to the global pattern and to ignore incongruent local features. Thus, this task may not have contributed to the executive-attention training. Thus, we might see better performance that generalizes if we expose monkeys to more than four intervention tasks that instate attention-control practice.

Second, it may be true that the animals had insufficient training even with the four intervention tasks that we used here. Significant negative correlations were generally seen between day-on-task and interference on the training tasks (Table 3). These effects were generally fit by a power function (although all of the variability appeared to be captured in the first few days of the training function); thus, it is possible that extensive overtraining would have produced more improvement, and perhaps given rise to generalize improvement. However, this is a delicate situation, in that overtraining builds associative cues that may indeed improve performance, but not in a way that generalizes to new conditions. That is, it is certainly true that the monkeys could be tested on the numerical Stroop task for sufficient trials that array size became the dominant response cue, such that behavior was controlled by array size and relatively unaffected by the extinguished numeral-meaning cue; in such a state, however, the animal could not switch flexibly and executively to “respond on numeral rather than array size” without interference. That is, one cannot improve the capacity to inhibit a strongly habitual response simply by building the associative strength of another stimulus-response pair. The question at hand is whether organisms can become better at controlling attention willfully, even in the face of strong, competing stimulus-control associations.

Third, it may of course be true that this is simply not possible—that monkeys (or children, or individuals with attention deficits, or…) cannot truly become more executive in attention control. This might certainly be true if one rejects the premise that cognitive control represents a different kind of processing than stimulus control. Whereas we acknowledge that behavior is frequently determined by low-level associations, automaticity, habits, and similar stimulus-driven factors, we agree with theorists who posit a higher-order, top-down, conceptual, effortful, goal-directed and intentional control. This is not to suggest that executive attention requires some dualist homunculus to serve as the executive, or to deny that these higher-order executive functions (e.g., inhibition, updating, metacognition, planning) emerge through experience. We simply suggest that the focus of attention—what stimuli get selected for processing and response—is determined at every moment by the competition between stimulus constraints, experiential constraints, and intentions or motivations, and that groups (including species, developmental stages, clinically defined populations) appear to differ with respect to the relative effectiveness of these executive-attention constraints.

Given this, it may still be that the relative potency of executive constraints is relatively fixed, stable, and resistant to training. Although training effects have been reported for young children, previous attention-training reports have been challenged. For example, Shalev *et al.* [22] used parent report to determine their outcomes, and whereas this measure is used often with great success, for this particular type of testing it could be prone to bias because parents naturally want their children to show improvement. Engle and his collaborators have urged caution in arguing for generalized training benefits, noting that improvement is typically limited to individual-task improvement, and that generalization of the constructs tested in cognitive training interventions has seldom been clear in any of the published literature [43,44,45].

Nevertheless, it has been reported that monkeys appear to become more attentive and less distractible as a result of initial joystick-task training [46], and so we certainly were optimistic that executive-attention could be improved in the present study. But perhaps the monkeys in the present research failed to show generalized benefits in attention control because they are already as good at executive attention as they can be, as a result of their extensive prior research and training histories. It is probably the case that generalized training effects are best manifest during critical periods of brain development and myelination (e.g., early childhood), or perhaps as treatments during periods of brain atrophy (e.g., dementia). Accordingly, it might be necessary to investigate whether generalized executive attention can be improved in younger (or much older) and relatively naïve animals, to test the possibility that generalized attention-training effects can indeed be found, but only early in the animals’ experience.

There were several other limitations to the current study. It should be noted that only four animals completed the research, from the original sample of seven rhesus monkeys, because three of the animals failed to reach pre-intervention criterion on the ANT assessment. Because the remaining animals took various numbers of trials—across months of testing—to reach the various performance criteria, it is impossible to disambiguate the type of training from the extent of training in the small sample.

Despite these limitations, this study advances our understanding of attention in several important ways. The absence of significant improvements may not show that significant, generalized training benefits are impossible, but the data do add to a growing corpus of results that challenge (or at least constrain) claims about the benefits of cognitive interventions. Moreover, they show the complexity of understanding attention and its control within a context of competing stimulus-control and cognitive-control variables. There are costs and benefits of training that emphasizes rigid, stimulus-response responding, and by the same token training that does improve executive attention might make the organism better able to deal flexibly with various response demands, but this will come at a cost to speed and effort. One might wonder under what circumstances a rhesus monkey—or a human child, for that matter—would find it adaptive to sacrifice efficiency and to increase effort in service of becoming more executive.

## Figures and Tables

**Table 1:** Study design, including pre/post assessments and training conditions.

Monkey = Obi	Luke	Han	Chewie
Pre-training attention network test assessment			
Attention Training	Control Training	Attention Training	Control Training
Attention network test assessment			
Control Training			Attention Training
Attention network test assessment			

**Table 2:** Performance means for accuracy and response time (RT) by task, test, and animal (pre = pre-training assessment; post = post-training assessment; postc = post-control assessment).

Task/Test	Baseline Accuracy	Congruent Accuracy	Incongruent Accuracy	Baseline RT (msec)	Congruent RT (msec)	Incongruent RT (msec)
Monkey = Obi						
ANT pre	97%	98%	97%	775	812	801
ANT post	98%	97%	99%	716	750	729
ANT postc	98%	96%	99%	634	702	664
Stroop	78%	83%	72%	593	607	635
Simon	88%	88%	71%	445	463	597
GLI	94%	92%	94%	1729	1730	1786
Monkey = Luke						
ANT pre	91%	90%	75%	787	928	919
ANT postc	97%	96%	97%	863	974	997
Monkey = Han						
ANT pre	77%	69%	51%	524	596	593
ANT post	83%	89%	54%	566	639	699
Stroop	85%	92%	69%	544	543	597
Simon	53%	52%	60%	240	270	350
GLI	97%	98%	97%	1714	1714	1752
Monkey = Chewie						
ANT pre	99%	96%	95%	748	805	858
ANT postc	98%	97%	94%	748	805	858
ANT post	97%	96%	96%	712	768	804
Stroop	86%	89%	84%	586	581	594
Simon	67%	79%	48%	443	430	543
GLI	69%	69%	67%	1646	1664	1604

**Table 3:** Significant correlations (* *p* < 0.05, ** *p* < 0.01) across animals between day-on-task and various performance indices of executive attention.

Task	Variables	Beta *
Stroop	Day of Testing and Difference in Accuracy (Baseline minus Incongruent)	−0.45 **
	Day of Testing and Difference in RT (Incongruent minus Baseline)	−0.32 *
	Day of Testing and Accuracy on Incongruent Trials	−0.60 **
Simon	Day of Testing and Difference in Accuracy (Baseline minus Incongruent)	−0.34 *
	Day of Testing and Difference in RT (Incongruent minus Baseline)	−0.31 *
	Day of Testing and Accuracy on Incongruent Trials	0.16
GLI	Day of Testing and Difference in Accuracy (Baseline minus Incongruent)	0.53 *
ANT	Day of Testing and Difference in Accuracy (Baseline minus Incongruent)	0.64 **
	Day of Testing and Difference in RT (Incongruent minus Baseline)	0.37 *
	Day of Testing and Accuracy on Incongruent Trials	−0.52 **
