# Bramlett-Parker, J.; Washburn, D.A. Can Rhesus Monkey Learn Executive Attention? *Behav. Sci.* 2016, *6*, 11

**DOI:** 10.3390/bs6040026

**Published:** 2016-11-29

**Authors:** Jessica Bramlett-Parker, David A. Washburn

**Affiliations:** Language Research Center, Department of Psychology, Georgia State University, Atlanta, GA 30302-5010, USA; bramlett1@student.gsu.edu

The authors wish to add the following correction to their paper published in *Behavioral Sciences* [1]:

“Monkey” in the title of the article “Can Rhesus Monkey Learn Executive Attention?” should be “Monkeys”.

The authors would like to apologize for any inconvenience caused to the readers by this change. The manuscript will be updated and the original will remain online on the article webpage.
